# Working Memory Predicts Long‐Term Recognition of Auditory Sequences: Dissociation Between Confirmed Predictions and Prediction Errors

**DOI:** 10.1111/sjop.13124

**Published:** 2025-05-21

**Authors:** L. Bonetti, E. Risgaard Olsen, F. Carlomagno, E. Serra, S. A. Szabó, M. Klarlund, M. H. Andersen, L. Frausing, P. Vuust, E. Brattico, M. L. Kringelbach, G. Fernández‐Rubio

**Affiliations:** ^1^ Center for Music in the Brain, Department of Clinical Medicine Aarhus University & the Royal Academy of Music Aarhus, Aalborg Denmark; ^2^ Centre for Eudaimonia and Human Flourishing Linacre College, University of Oxford Oxford UK; ^3^ Department of Psychiatry University of Oxford Oxford UK; ^4^ Department of Education, Psychology, Communication University of Bari Bari Italy; ^5^ Department of Psychology and Behavioural Sciences Aarhus University Aarhus Denmark

**Keywords:** aging, long‐term memory, musical training, predictive coding, working memory

## Abstract

Memory is a crucial cognitive process involving several subsystems: sensory memory (SM), short‐term memory (STM), working memory (WM), and long‐term memory (LTM). While each has been extensively studied, the interaction between subsystems, particularly in relation to predicting temporal sequences, remains largely unexplored. This study investigates the association between WM and LTM, and how these relate to aging and musical training. Using three datasets with a total of 243 healthy volunteers across various age groups, we examined the impact of WM, age, and musical training on LTM recognition of novel and previously memorized musical sequences. Our results show that WM abilities are positively associated with the identification of novel sequences, but not with the recognition of memorized sequences. Additionally, musical training has a similar positive impact on the identification of novel sequences, while increasing age is associated with reduced memory performance. Different cognitive processes are involved in handling prediction errors compared to confirmatory predictions, and WM contributes to these processes differently. Future research should extend our investigation to populations with memory impairments and explore the underlying neural substrates.

**Summary**

- The working memory (WM) capacity is positively associated with long‐term memory (LTM) skills, particularly the identification of novel musical sequences in an old/new auditory recognition task.
- Musical training contributes to an increase, while aging is associated with a decrease, in LTM performance.
- Different cognitive processes underlie prediction errors versus confirmatory predictions, with WM playing distinct roles in each.

## 1 Introduction

Memory is a fundamental cognitive process that enables the storage, retrieval, and use of information once the original input is no longer present (LaVoie and Cobia 2007; Squire 2009; Sridhar et al. 2023; Tulving and Craik 2005; Zlotnik and Vansintjan 2019). Memory is typically divided into three key subsystems, each serving distinct functions while interacting with the environment. These subsystems are (1) sensory memory (SM) (Luck and Hollingworth 2008; Pearson and Brascamp 2008) and short‐term memory (STM) (Alvarez and Cavanagh 2004; Cowan 2001), (2) working memory (WM) (Baddeley 2020; Cowan et al. 2020; Hitch and Baddeley 1976), and (3) long‐term memory (LTM) (Postman et al. 1975; Shiffrin and Atkinson 1969; Unsworth 2016).

SM briefly retains sensory data after the stimulus has been removed (Bonetti et al. 2022; Garrido et al. 2009; Luck and Hollingworth 2008; Näätänen et al. 2005; Pearson and Brascamp 2008), providing the opportunity for this data to be transferred to STM, which is a limited‐capacity storage system that maintains information consciously for short periods of time (Alvarez and Cavanagh 2004; Cowan 2001). A common example of STM is remembering a phone number just long enough to write it down.

WM is a more complex and dynamic memory subsystem, originally proposed by Baddeley and Hitch in 1974 and refined in subsequent works (Baddeley 2020; Hitch and Baddeley 1976). It is defined as a limited‐capacity system responsible for the temporary storage and manipulation of information, a process which is necessary for complex tasks such as comprehension, learning, and reasoning. WM has been strongly linked to general cognitive abilities (Conway et al. 2003), with its distinguishing feature being the active manipulation of information, unlike STM, which functions primarily as a temporary storage system and does not entail manipulating information (Alvarez and Cavanagh 2004; Cowan 2001). The development of the WM concept originally arose from the limitations of the modal model of memory, which was prevalent at the time but failed to account for the dynamic nature of cognitive processing. In contrast to this earlier model, the embedded‐processes approach views WM as an executive system that temporarily activates LTMs, thereby enabling real‐time manipulation of information (Cowan et al. 2020).

LTM is the subsystem responsible for storing information over extended periods, ranging from hours to months, and in some cases, throughout an individual's entire life (Smith et al. 2005). LTM encompasses episodic (Horzyk et al. 2017), procedural (Müller et al. 2016), and semantic memory (Horzyk et al. 2017), which relate to personal life events, motor skills, and factual knowledge, respectively. Based on the conscious availability of stored information, LTMs are classified into two main types: procedural (implicit) and declarative (explicit) memory (Cohen and Squire 1980). Procedural memory underlies a variety of skills, such as learning how to ride a bike, which are performed without conscious effort (Squire and Zola 1996). In contrast, declarative memory refers to consciously accessible information that can be actively recalled and verbalized (Squire and Zola 1996). Before information can be stored in LTM, it must first be encoded. Encoding is the process of transforming sensory input into a format suitable for long‐term storage (Evans 2017; Tversky 1973). This transformation can occur through various modalities, such as visual, acoustic, or semantic, depending on the nature of the information and its associated meanings. Once encoded, information becomes accessible for retrieval or recognition. The process of encoding is closely linked to SM, STM, and WM, as information is initially perceived through the senses, briefly held in STM or manipulated by WM, and ultimately encoded into LTM for long‐term storage (Winkler and Cowan 2005).

Once a memory is consolidated in LTM, it can be accessed and utilized through two primary methods: recall and recognition (Postman et al. 1975). Recall involves retrieving information from memory, either without any prompts or with cues that are associated with the information (Pan et al. 2019). In contrast, recognition involves identifying specific information as having been previously encountered (Postman et al. 1975). The foundational dual‐process framework by Anderson and Bower (1972) suggests that recall and recognition are supported by distinct cognitive processes, each with its own set of steps to reach a conclusion. This distinction aligns with findings such as those of Tversky (1973), who showed that encoding strategies differ when individuals anticipate a recall test versus a recognition test. When comparing the two, research by Postman et al. (1975) demonstrated that performance on recall tests declines more rapidly than on recognition tests. Recent studies have further corroborated this pattern (Cleary 2018; Furey et al. 2023). Within recognition, the literature further differentiates between two distinct processes: familiarity and recollection (Mandler 1980). The key difference lies in context dependence—recollection requires a semantic context to properly place the retrieved information, whereas familiarity does not. As a result, recollection tends to exhibit slower forgetting rates compared to familiarity (Yonelinas and Levy 2002).

There is also evidence that the mechanisms for recognizing previously encountered information differ from those involved in recognizing novel or unknown stimuli (Bonetti et al. 2023; Wiser et al. 2000). This distinction is primarily supported by neural evidence, suggesting that the recognition of novelty, or the detection of something unfamiliar, is mediated by processes such as prediction error (Bonetti et al. 2023; Bonetti, Fernández‐Rubio, Lumaca, et al. 2024; Bonetti, Fernández‐Rubio, Carlomagno, et al. 2024; Pupillo et al. 2023). Recently, we explored this phenomenon through a series of behavioral and neuroscientific studies focused on LTM recognition of a specific type of stimulus: musical sequences. These stimuli are unique because they generate meaning through their combination and progression over time, which complies not only with biological constraints but also with the aesthetic conventions of a specific musical culture (Koelsch et al. 2019; Vuust et al. 2022). Musical sequences differ from more static stimuli traditionally used in memory research and from auditory sequences consisting of random time patterns, where no emotional or cognitive meaning emerges through their progression (Bale et al. 2017; Campo and Brattico 2022; Kang et al. 2017, 2018). Our findings revealed that individuals could memorize a musical piece with remarkable ease, even after just a few repetitions. Moreover, participants were able to recognize short melodies extracted from the original piece, as well as identify novel melodies or variations of the original sequences. Collectively, these studies suggest that musical sequences offer an effective model for studying memory processes related to sequences that unfold over time and that recognition of novel versus previously memorized sequences is overall more challenging (Bonetti et al. 2023; Bonetti, Fernández‐Rubio, Lumaca, et al. 2024; Bonetti, Fernández‐Rubio, Carlomagno, et al. 2024; Fernández‐Rubio, Carlomagno, et al. 2022; Fernández‐Rubio, Brattico, et al. 2022).

Among the various factors that can influence the memory subsystems described above, aging is one of the most well documented, with numerous studies highlighting that memory is among the cognitive functions most susceptible to decline with advancing age (Bender and Raz 2012; Cabeza et al. 2018; Clapp and Gazzaley 2012; Dahan et al. 2020; Ochsner and Kosslyn 2013; Small 2001). This is especially true for declarative memory, which tends to deteriorate more rapidly (Ofen and Shing 2013; Shing et al. 2023). While this decline is a natural process, certain conditions can accelerate it (Small 2001; Squire and Zola 1996). Importantly, the effects of aging are not limited to LTM (Luo and Craik 2008; Ochsner and Kosslyn 2013); they also impact WM (Kirova et al. 2015; Wang et al. 2011), which plays a critical role in complex cognitive tasks. Gordon‐Salant and Cole (2016) provide evidence of this by comparing the speech recognition abilities of younger and older adults, further categorizing them by their WM capacity. This study found that both age and WM capacity affect speech recognition, with older adults showing greater difficulty in recognizing sentences, particularly those with lower WM capacity. The interaction between age and WM was significant, suggesting that age‐related decline in WM exacerbates difficulties in tasks requiring memory and real‐time processing, such as sentence recognition (Bianco and Chait 2023; Holmes and Griffiths 2019; Lad et al. 2020). Interestingly, recent findings indicate that, while aging does not necessarily impair the LTM recognition of familiar musical melodies, older adults perform worse than younger adults when it comes to recognizing variations of these melodies (Bonetti, Fernández‐Rubio, Lumaca, et al. 2024). This suggests that, although basic recognition abilities may be preserved with age, the ability to adapt to changes or variations in learned sequences declines, potentially due to reduced WM capacity or changes in cognitive flexibility.

While extensive research has been conducted on both WM (Baddeley 2020; Cowan et al. 2020; Hitch and Baddeley 1976) and LTM (Postman et al. 1975; Shiffrin and Atkinson 1969; Unsworth 2016), much remains to be learned about the interaction between the two and the underlying mechanisms that connect them. The two pioneering models of WM, as previously discussed, provide different perspectives on the relationship between WM and LTM. In Baddeley's model (Baddeley 2020), the central executive component of WM accesses information stored in LTM and uses it to guide present actions. In contrast, Cowan's approach (Cowan et al. 2020) conceptualizes WM as a temporary activation of LTM, serving as a “window to the present”. Despite these differences, both models agree that WM enables individuals to respond to environmental stimuli by integrating incoming information with stored knowledge from LTM. This capacity to manipulate and integrate information is key to why WM has been strongly linked to general cognitive abilities (Conway et al. 2003) and why it remains a crucial variable in memory research. Although there is theoretical and empirical support for the interaction between these two memory systems, our understanding of the precise nature of this relationship remains limited.

The association between LTM and WM seems particularly relevant when examining how WM influences various types of recognition and predictive processes (Auksztulewicz and Friston 2016; Friston 2012b). For example, one area of interest is the relationship between WM and the ability to recognize previously memorized information, which depends on the accuracy of predictions about expected outcomes (e.g., the predicted incoming sound in a temporal sequence) compared to the actual outcomes (e.g., the actual incoming sound in the sequence). Similarly, it is relevant to understand how WM relates to the recognition of variations within a sequence, involving prediction error processes (e.g., when the incoming sound in the sequence deviates from the predicted sound). Previous studies showed that the acquisition of auditory memory for temporal sequences takes place via repeated exposure (Agus et al. 2010; Agus and Pressnitzer 2021; Kang et al. 2017, 2018; Ringer et al. 2022) and is impacted by aging (Bianco and Chait 2023). Understanding the dynamics of LTM and WM is therefore crucial when dealing with sequential information that evolves over time, where meaning is constructed progressively (as in the example of the temporal sequences of sounds). The gap in our knowledge about how WM interacts with these processes underscores the need for further investigation into how memory systems handle both expected and unexpected changes in sequential patterns.

Building on our extensive research into long‐term encoding and recognition of musical sequences (Bonetti et al. 2021, 2023; Bonetti, Fernández‐Rubio, Lumaca, et al. 2024; Bonetti, Fernández‐Rubio, Carlomagno, et al. 2024; Fernández‐Rubio, Carlomagno, et al. 2022; Fernández‐Rubio, Brattico, et al. 2022; Fernández‐Rubio et al. 2024), the current study investigates how WM and LTM interact. Our aim was to characterize the relationship between these two memory systems, particularly with regard to recognition and predictive processes. To this end, we employed auditory memory tasks, where brief musical sequences were repeated and altered to probe different LTM processes, and a widely used measure of WM across three distinct datasets, involving 243 healthy participants. To complement our analyses, we examined the effects of aging and musical training on LTM, drawing on established findings in the field (Criscuolo et al. 2019, 2022; Fernández‐Rubio et al. 2024; Losch et al. 2024; Roman‐Caballero et al. 2018; Talamini et al. 2017).

## 2 Methods

### 2.1 Participant Samples

We collected demographic and behavioral data from three different samples for a total of 244 participants at three time points: 2021, 2022, and 2024.

The 2021 dataset consisted of 83 participants (49 females) aged 18–63 years old (mean age: 28.74 ± 8.10 years). Participants came from Western countries and had homogeneous educational backgrounds. The project was approved by the Institutional Review Board (IRB, case number: DNC‐IRB‐2020‐006). Independent neuroscience results obtained from this dataset have been published in a previous work (Bonetti, Fernández‐Rubio, Carlomagno, et al. 2024).

The 2022 dataset comprised 77 participants (43 females) aged 18–81 years (mean age: 45.58 ± 23.31 years). All participants were Danish and had homogeneous educational backgrounds. The project was approved by the IRB (case number: DNC‐IRB‐2021‐012). Neuroscientific and behavioral results obtained from this dataset have been published in two previous papers (Bonetti, Fernández‐Rubio, Lumaca, et al. 2024; Fernández‐Rubio et al. 2024).

The 2024 dataset included 84 participants (51 females) aged 18–83 years (mean age: 45.08 ± 24.25 years). Participants presented homogeneous educational backgrounds. The project was approved by the Ethics Committee (ref. 1‐10‐72‐127‐23).

The following inclusion criteria were applied to all datasets: (1) normal health (no reported neurological nor psychiatric disease), (2) normal hearing (non‐pathological age‐related hearing decline was not an exclusion criterium if participants could comfortably perform the task), (3) normal sight or corrected normal sight, (4) individuals aged over 18 years and (4) understanding and acceptance of experimental procedure. All experimental procedures complied with the Declaration of Helsinki—Ethical Principles for Medical Research, and informed consent was obtained from all participants before starting the study.

### 2.2 Long‐Term Memory Measure

We employed the old/new auditory recognition task we have used in our previous studies (e.g., Bonetti, Fernández‐Rubio, Carlomagno, et al. 2024; Fernández‐Rubio, Brattico, et al. 2022; Fernández‐Rubio et al. 2024) to measure LTM. The task consists of an encoding and a recognition phase. During the encoding phase, participants listened to a shortened version of the right‐hand part of J. S. Bach's Prelude No. 2 in C minor, BWV 847, and were instructed to memorize it to the best of their ability. The piece had a duration of 25 s and was presented twice. Afterwards, during the recognition phase, participants listened to fragments of the prelude (i.e., memorized sequences) and variations of these fragments (i.e., novel sequences) and were requested to state whether each sequence was memorized or novel using a response pad. All sequences had a duration of 1750 ms and were presented in random order.

Memorized sequences (M) consisted of the first five tones of the first three measures of the musical piece, while novel sequences (N) were created through systematic variations of the three M sequences. This procedure consisted of changing every musical tone of the sequence after the first (NT1), second (NT2), third (NT3), or fourth (NT4) tone (see Bonetti, Fernández‐Rubio, Carlomagno, et al. 2024 for further details). Due to scientific and time constraints, the number and type of trials during the recognition phase of the task differed between the three datasets. For example, participants in the 2021 and 2022 datasets were presented with 27 M trials (3 sequences × 9 repetitions) and 27 NT1 trials, while participants in the 2024 dataset listened to 42 M trials (3 sequences × 14 repetitions) and were not presented with NT1 trials. Importantly, the experimental procedure was identical in all three data collections, ensuring that the old/new auditory recognition task probed LTM abilities, albeit with differences in the task design. Count scores (i.e., number of correct responses out of total number of trials) were obtained for each stimulus category. The anonymized data can be found in Table S1.

### 2.3 Working Memory Measure

WM skills were estimated using the Working Memory Index from the Wechsler Adult Intelligence Scale IV (Wechsler 2009). This measure comprises two subsets: Digit Span, where participants listen to sequences of numbers and reproduce them orally in the same, reversed, or ascending order, and Arithmetic, where they listen to mathematical problems and provide solutions orally without external aids. We combined the raw scores from the Digit Span and Arithmetic subtests to provide a total working memory value. Scores ranged from 5 to 70.

### 2.4 Musical Training Measure

We used the musical training facet from the Goldsmiths Musical Sophistication Index questionnaire (Müllensiefen et al. 2014) to measure participants' history of formal musical training. This part of the questionnaire comprises seven questions related to self‐assessed musicianship and extent of musical training and practice. Each item was rated on a 7‐point Likert scale, with total scores ranging from 7 (i.e., no history of formal musical training) to 49 (i.e., professional musicians). In the case of datasets from 2022 and 2024, we employed the Danish version of the questionnaire (Møller et al. 2024).

### 2.5 Statistical Analysis

Generalized linear models (GLMs) were used to assess the association between WM and LTM skills. Two models were computed, one for LTM for M sequences (LTM‐M) and one for LTM for N sequences (LTM‐N), with WM, musical training, and age as predictors. The GLMs were estimated independently for each dataset to account for heterogeneity in task design and to distinguish dataset‐specific effects from robust effects observed across datasets.

A binomial regression model with a logit link function was employed to capture the discrete nature of the dependent variables, as both LTM‐M and LTM‐N represent discrete distributions indicating the probability of giving a correct response (*n*) out of the total number of responses (*k*) (Theobald et al. 2019). The GLMs were computed at *α* = 0.008 after applying the Bonferroni correction (0.05 divided by 6 models, two per dataset). Effect displays were created for the predictor of interest (WM) while keeping the values of other predictors fixed (Fox 2003; Fox and Weisberg 2018).

Additional analyses were conducted to further examine the relationship between WM skills and detection of prediction errors. Binomial regression models with a logit link function were computed with WM, musical training, and age as predictors, and different categories of N (NT1, NT2, NT3, and NT4) as outcome variables. These analyses were conducted independently for each dataset.

All analyses were computed in R version 4.4.3 (RStudio Team 2020).

## 3 Results

One participant was removed from the analysis of the 2024 dataset due to missing data, bringing the final sample to *N* = 83 (see Table 1). Two GLMs were computed for each dataset to assess the relationship between the predictors (WM, age, and musical training) and LTM‐M, and between the predictors and LTM‐N.

**TABLE 1:** Data description.

Variable	2021 (*N* = 83)				2022 (*N* = 77)				2024 (*N* = 83)			
	Mean	Min.	Max.	*k*	Mean	Min.	Max.	*k*	Mean	Min.	Max.	*k*
LTM‐M	22.33	6	27	27	22.91	2	27	27	36.3	14	42	42
LTM‐N	82.64	29	108	108	45.05	0	54	54	65.67	25	84	84
WM	39.86	27	55	—	40.47	26	55	—	41.02	21	57	—
Age	28.74	18	63	—	45.58	18	81	—	45.08	18	83	—
Musical training	19.60	0	41	—	23.19	6	41	—	22.36	10	38	—

Abbreviations: *k*, number of trials; LTM‐M, long‐term memory, memorized sequences; LTM‐N, long‐term memory, novel sequences; WM, working memory.

**FIGURE 1:**
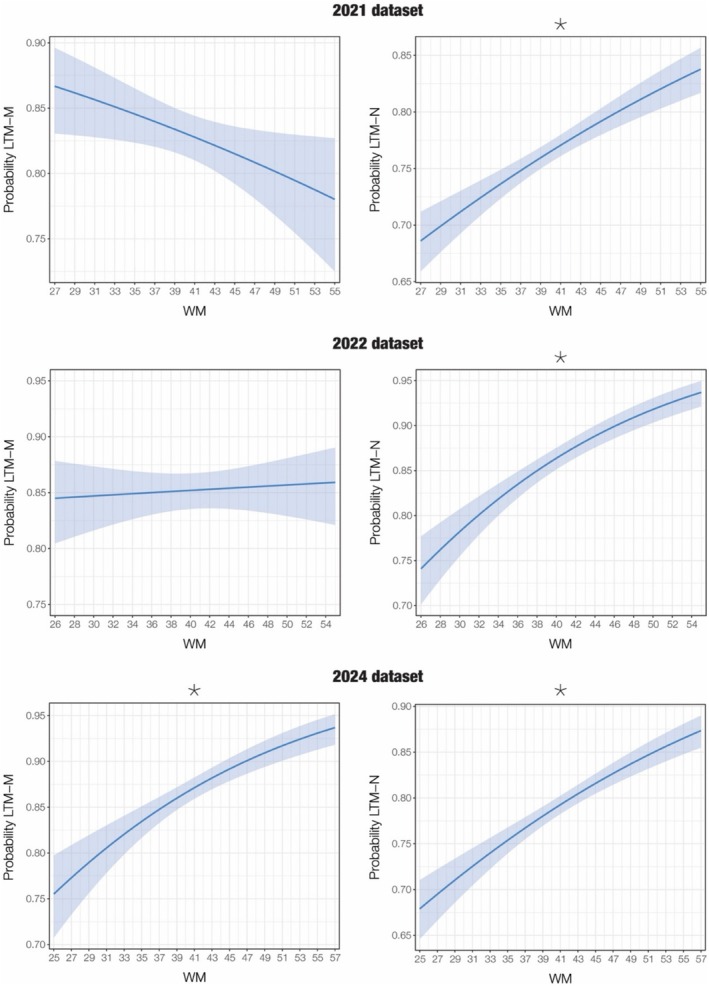
Relationship between working memory (WM) and long‐term memory (LTM) abilities. The plots show the predicted probability of a correct response in LTM for memorized sequences (LTM‐M) and LTM for novel sequences (LTM‐N) as a function of WM, based on a binomial regression model with a logit link. The solid line represents the model's predicted values for accuracy across the range of WM scores, while the shaded area reflects the 95% confidence interval. Predictions are adjusted for age and musical training, which are held constant at their mean value. Asterisk (*) denotes statistical significance at the Bonferroni‐corrected *p* < 0.008 level.

In relation to the 2021 dataset, musical training significantly contributed to higher scores in LTM recognition of both M sequences and N sequences. In addition, WM was positively associated with LTM‐N, as the odds of correctly identifying an N sequence increased with higher WM abilities. The results for both GLMs are reported in Table 2 and Figure 1.

**TABLE 2:** Generalized linear regression models (binomial, logit link) from the 2021 dataset.

LTM	Predictor	*β*	SE	*z*	*p*
M	(Intercept)	1.239	0.445	2.782	0.005*
	WM	−0.022	0.010	−2.245	0.025
	Age	0.004	0.008	0.545	0.586
	Musical training	0.057	0.008	7.125	< 0.0001*
N	(Intercept)	−0.524	0.198	−2.647	0.008
	WM	0.031	0.005	6.805	< 0.0001*
	Age	−0.009	0.003	−2.743	0.006*
	Musical training	0.038	0.003	11.216	< 0.0001*

Abbreviations: *β*, regression coefficient; LTM, long‐term memory; M, memorized sequences; *N*, novel sequences; SE, standard error; WM, working memory.

* Statistical significance at the Bonferroni‐corrected *p* < 0.008 level.

Regarding the 2022 dataset, no predictor variables were significantly associated with LTM recognition of M sequences. Conversely, the model which included LTM‐N showed that both WM and musical training contributed to an increase, while age contributed to a decrease in accuracy for LTM‐N, as shown in Table 3 and Figure 1.

**TABLE 3:** Generalized linear regression models (binomial, logit link) from the 2022 dataset.

LTM	Predictor	*β*	SE	*z*	*p*
M	(Intercept)	1.391	0.442	3.148	0.002*
	WM	0.004	0.009	0.436	0.663
	Age	−0.007	0.003	−2.354	0.019
	Musical training	0.023	0.010	2.409	0.016
N	(Intercept)	−0.122	0.323	−0.378	0.705
	WM	0.057	0.007	8.347	< 0.0001*
	Age	−0.024	0.002	−10.441	< 0.0001*
	Musical training	0.035	0.007	5.027	< 0.0001*

Abbreviations: *β*, regression coefficient; LTM, long‐term memory; M, memorized sequences; *N*, novel sequences; SE, standard error; WM, working memory.

* Statistical significance at the Bonferroni‐corrected *p* < 0.008 level.

With regards to the 2024 dataset, WM was significant and contributed to higher accuracy scores in both LTM for M sequences and for N sequences (see Table 4 and Figure 1). Musical training was also significant and contributed to higher scores in both LTM for M sequences and for N sequences. Finally, older age was associated with lower accuracy in LTM for N sequences.

**TABLE 4:** Generalized linear regression models (binomial, logit link) from the 2024 dataset.

LTM	Predictor	*β*	SE	*z*	*p*
M	(Intercept)	−0.627	0.339	−1.851	0.064
	WM	0.049	0.008	6.402	< 0.0001*
	Age	−0.002	0.002	−1.090	0.276
	Musical training	0.028	0.009	3.124	0.002*
N	(Intercept)	0.616	0.196	3.149	0.002*
	WM	0.037	0.004	8.236	< 0.0001*
	Age	−0.014	0.001	−11.221	< 0.0001*
	Musical training	−0.006	0.005	−1.266	0.206

Abbreviations: *β*, regression coefficient; LTM, long‐term memory; M, memorized sequences; *N*, novel sequences; SE, standard error; WM, working memory.

* Statistical significance at the Bonferroni‐corrected *p* < 0.008 level.

### 3.1 Additional Analyses

To further explore the association between WM and prediction error processes, a set of additional GLMs was computed. The models comprised the same predictors (WM, age, and musical training) and different categories of LTM‐N (NT1, NT2, NT3, NT4). A binomial model with a logit link function was employed, and the models were estimated independently for each dataset, as the stimulus categories and number of trials differed between them.

In all cases, WM was significant and contributed to higher scores in the different categories of LTM‐N: NT1 (*p* = 0.011), NT2 (*p* < 0.0001), NT3 (*p* = 0.0002), and NT4 (*p* = 0.0003) in the 2021 dataset; NT1 (*p* < 0.0001) and NT3 (*p* < 0.0001) in the 2022 dataset; and NT3 (*p* < 0.0001) and NT4 (*p* < 0.0001) in the 2024 dataset. Musical training was also significant and contributed to higher scores in LTM for all N categories in the 2021 dataset (*p* < 0.0001), NT1 (*p* = 0.007) and NT3 sequences (*p* = 0.0004) in the 2022 dataset, and NT3 sequences (*p* = 0.002) in the 2024 dataset. Finally, age showed a significant negative association with LTM for NT4 sequences (*p* < 0.0001) in the 2021 dataset, both NT1 (*p* < 0.0001) and NT3 sequences (*p* < 0.0001) in the 2022 dataset, and both NT3 (*p* < 0.0001) and NT4 sequences (*p* < 0.0001) in the 2024 dataset. The results of these analyses are reported in Table S2.

## 4 Discussion

The results from three different datasets revealed a significant, positive relationship between WM and LTM. These findings strengthen the concept that memory subsystems are interconnected, aligning with previous models that link WM to LTM (Baddeley 2020; Cowan et al. 2020; Unsworth 2010, 2016). Notably, this relationship is observed specifically in the identification of novel sequences, suggesting that the role of WM varies between recognizing previously memorized sequences—where predictions are confirmed—and identifying new melodies—where predictions are disrupted, generating prediction errors. This distinction implies that these two LTM processes may operate differently. These findings are consistent with our prior neuroscientific studies, which showed that different neural processes underpin the recognition of memorized versus novel musical sequences (Bonetti et al. 2021, 2023; Bonetti, Fernández‐Rubio, Lumaca, et al. 2024; Bonetti, Fernández‐Rubio, Carlomagno, et al. 2024; Fernández‐Rubio, Carlomagno, et al. 2022; Fernández‐Rubio, Brattico, et al. 2022; Fernández‐Rubio et al. 2024). The strong association between WM and the recognition of novel melodies indicates that WM is closely related to prediction error and plays a role in detecting discrepancies. Conversely, WM does not appear to be as involved in the recognition of previously memorized sequences, as it was only significant in one of the three datasets, suggesting that this process may not engage WM to the same extent.

Predictive processes are a fundamental concept in psychology and neuroscience, leading to the formulation of the Predictive Coding Theory (PCT), which has been extensively reviewed by several authors (Auksztulewicz and Friston 2016; Friston and Kiebel 2009; Friston 2012b, 2019; Heilbron and Chait 2018; Hodson et al. 2024). In particular, predictions can be confirmed or violated, generating prediction errors in the latter case. Den Ouden et al. (2012) offer a comprehensive overview of distinct types of prediction error, outlining their functions and neural underpinnings. They categorize prediction errors into sensory (e.g., oddball paradigm), cognitive (when an event deviates from expectations), and motivational (when value is assigned to an outcome). Despite their different functions and contexts, these types all share a common mechanism of comparing top‐down knowledge with bottom‐up input. This highlights that prediction error is a core mechanism present across both lower and higher‐order brain regions and cognitive processes. Our study aligns with this framework, focusing on cognitive prediction error during the recognition of varied musical sequences. In this task, participants consciously evaluated and predicted the upcoming sounds based on previously heard sequences. The introduction of novel sounds created a cognitive prediction error, as the anticipated sound did not match the actual event. Our findings demonstrate that WM abilities influence performance in tasks involving such cognitive prediction errors, thereby affecting the accuracy of these predictions.

Additional research linking prediction error to WM and LTM underscores the importance of their interaction in memory consolidation and reconsolidation (Fernández et al. 2016). In this context, prediction error serves not only as a mechanism for detecting discrepancies but also as a facilitator of learning. Specifically, when there is a mismatch between encoded and experienced information, the process involves activating the memory, destabilizing it through the prediction error, and then updating and reinforcing it. WM plays a crucial role in this process by enhancing the flexibility and efficiency of information updating. This strategy for cognitive updating is supported by several theoretical frameworks, including PCT (Barron et al. 2020; Bubic et al. 2010) and the Bayesian Brain Hypothesis (Friston 2012a), which outline its neural mechanisms and behavioral characteristics.

Our recent studies on the neurophysiology of music encoding and recognition also provide compelling evidence for the relationship between WM and LTM. For example, we demonstrated that WM abilities influence the brain mechanisms involved in encoding single sounds. Specifically, individuals with higher WM exhibited greater recruitment of frontal regions, such as the right frontal operculum, during sound encoding (Bonetti et al. 2021). In terms of LTM recognition of music, WM was linked to enhanced brain responses when recognizing previously memorized sounds (Fernández‐Rubio, Carlomagno, et al. 2022). This finding was further supported and expanded by our recent work, which showed that WM is associated with both the recognition of previously memorized and the identification of varied melodies, with a particularly strong effect for varied melodies. This effect is notably related to the prediction error signal generated by these variations (Bonetti, Fernández‐Rubio, Carlomagno, et al. 2024). Interestingly, this result was corroborated by another study in which we found that WM abilities had a more pronounced impact on the prediction error signal compared to age, revealing similar prediction error signals in older adults with high WM and young adults with low WM (Bonetti, Fernández‐Rubio, Lumaca, et al. 2024). Moreover, our earlier research identified a relationship between enhanced mismatch negativity (MMN)—a brain signal indicative of automatic or sensory prediction error—and higher WM abilities (Bonetti et al. 2018). This finding is significant as it extends the association between prediction error and WM beyond LTM, implicating a brain signal often interpreted as reflecting automatic sensory memory processes.

In the current study, LTM capacity for identification of novel sequences was significantly influenced by participants' age. Consistent with our recent findings (Bonetti, Fernández‐Rubio, Lumaca, et al. 2024; Fernández‐Rubio et al. 2024), we observed that older adults had no significant difficulty recognizing previously memorized melodies, but their ability to recognize varied melodies was notably impaired. Although we did not specifically test for an interaction between WM skills and age, these results could suggest that the association between WM and LTM becomes more critical as cognitive decline progresses with age, making tasks like recognizing novel sequences more challenging. For younger participants, the relationship might be masked by a ceiling effect, where WM and LTM are already functioning optimally. In this context, older adults might rely more heavily on their WM resources to compensate for difficulties in recognizing novel sequences, potentially indicating that they allocate more WM resources to handle prediction errors in LTM tasks.

Consistent with our findings, existing literature acknowledges a natural, non‐pathological cognitive decline associated with aging, though this decline is not necessarily exacerbated by medical conditions (Dahan et al. 2020; Gordon‐Salant and Cole 2016). Studies often highlight episodic (or autobiographical) memory as the primary domain affected by aging, while semantic memory tends to remain relatively intact (Ofen and Shing 2013; Shing et al. 2023). Shing et al. (2023) theorize that this asymmetry leads to difficulties in processing novel information for older adults. This reliance on well‐established semantic knowledge might hinder the ability to form new episodic memories efficiently. In our study, the LTM task used, which involves translating sounds into mental (musical) representations, is arguably more aligned with semantic memory than episodic memory. This may explain why older participants did not significantly underperform compared to younger participants in recognizing previously memorized sequences. Additionally, older adults are more susceptible to retroactive interference during recognition tasks, especially when faced with distractors (Clapp and Gazzaley 2012; Solesio‐Jofre et al. 2012). Their neural activation patterns diverge from those of younger individuals, and they show reduced neural representation of prediction errors in feedback‐driven reinforcement learning, although evidence regarding confirmatory prediction abilities remains inconclusive (Eppinger et al. 2013; Samanez‐Larkin et al. 2014). Our results further support a dissociation between the processes involved in prediction error for novel sequences and the recognition of previously learned information, with this dissociation becoming more pronounced in aging populations. As already mentioned above, this aligns with our recent findings on age‐related changes in brain dynamics during music recognition (Bonetti, Fernández‐Rubio, Lumaca, et al. 2024). We observed that, while prediction error signals decrease with healthy aging, recognition of previously memorized sequences is associated with compensatory brain mechanisms, such as increased activity in the left auditory cortex. This increase likely compensates for reduced functioning in memory‐related regions of the medial temporal lobe, such as the hippocampus and inferior temporal cortex. The absence of such compensatory mechanisms in older adults corresponds with the reduced behavioral performance observed across datasets.

Finally, our study found a positive association between musical training and memory performance. This finding supports previous research that associates musical expertise with enhanced auditory, musical, and general cognitive abilities, as well as increased neural responses during these tasks (Criscuolo et al. 2019, 2022; Losch et al. 2024; Roman‐Caballero et al. 2018; Schellenberg 2005, 2011; Schulze and Tillmann 2013; Talamini et al. 2017). For example, Schulze and Tillmann (2013) explored forward and backward recognition of different types of auditory information and found a positive correlation between WM for pitch sequences—encompassing both recognition and manipulation of these sequences—and years of musical training. Their findings indicated that increased years of musical training were associated with better performance on tasks involving pitch sequence manipulation, suggesting a higher domain‐specific capacity linked to musical expertise. In a meta‐analysis of 29 studies, Talamini et al. (2017) demonstrated that musicians perform better than nonmusicians in memory tasks, particularly those related to short‐term and working memory. Our own research also supports this, as demonstrated in a study comparing memory functions in musicians and amateurs versus nonmusicians (Criscuolo et al. 2019). The underlying reasons for the positive association between musical training and cognitive functioning remain nonetheless debated. Schellenberg (2020) recently proposed that the relationship might stem from a higher likelihood of individuals with higher intelligence engaging in musical training, rather than musical training directly enhancing cognitive abilities. This challenges the earlier hypotheses that musical training itself improves cognitive abilities (Schellenberg 2005, 2011). While further research is needed to clarify this relationship, the results from our current study corroborate the positive association between musical expertise and memory abilities.

To conclude, our findings reveal that WM capacity is significantly associated with the identification of novel sequences but is not similarly related to the recognition of previously memorized sequences. This suggests that different cognitive processes are involved in handling confirmatory predictions versus prediction errors. Benefitting from large sample sizes and replication across multiple datasets, our study significantly advances the understanding of the relationship between WM and LTM, highlighting how these subsystems are influenced by aging and musical training. However, as our study primarily identifies positive relationships between different memory subsystems, further research is needed to elucidate the specific mechanisms through which WM arguably impacts LTM processes. Furthermore, exploring this association in populations with memory impairments could yield additional valuable insights.

## Author Contributions

L.B. and G.F.‐R. conceived the hypotheses. L.B., G.F.‐R., E.B., and M.L.K. designed the study. L.B., G.F.‐R., M.L.K., E.B., and P.V. recruited the resources for the experiment. L.B., G.F.‐R., E.R.O., F.C., E.S., M.K., M.H.A., and L.F. collected the data. G.F.‐R. performed statistical analyses, with the contribution of L.B. E.B., M.L.K. and P.V. provided essential help to interpret and frame the results within the psychological and neuroscientific literature. L.B., G.F.‐R., and S.A.S. wrote the first draft of the manuscript. G.F.‐R. prepared the figures. All the authors contributed to and approved the final version of the manuscript.

## Disclosure

Code Availability: The code used for computing descriptive statistics and plotting results is freely available on GitHub: https://github.com/gemmaferu/interactions‐ltm‐wm.

## Conflicts of Interest

The authors declare no conflicts of interest.

## Supporting information


**Table S1.** Anonymized data. This table presents the anonymized data used in this study. The table is organized in the following columns: participant number (“id”); dataset (“dataset”); participant age (“age”); combined raw scores from the Digit Span and Arithmetic subtests from the Working Memory Index in Wechsler Adult Intelligence Scale IV (“WM”); score from the musical training facet from the Goldsmiths Musical Sophistication Index questionnaire (“musictraining”); number of correct responses (“mem_n”) and total number of trials (“mem_k”) for memorized sequences in the old/new auditory recognition task; number of correct responses (“nov_n”) and total number of trials (“nov_k”) for novel sequences in the old/new auditory recognition task; number of correct responses (“novt1_n”) and total number of trials (“novt1_k”) for novel T1 sequences in the old/new auditory recognition task; number of correct responses (“novt2_n”) and total number of trials (“novt2_k”) for novel T2 sequences in the old/new auditory recognition task; number of correct responses (“novt3_n”) and total number of trials (“novt3_k”) for novel T3 sequences in the old/new auditory recognition task; and number of correct responses (“novt4_n”) and total number of trials (“novt4_k”) for novel T4 sequences in the old/new auditory recognition task.

**Table S2.** Relationship between working memory and detection of prediction errors. This table presents the results of statistical analyses regarding the relationship between working memory (WM) and the detection of prediction errors. Binomial regression models with a logit link were computed independently for each dataset. The models comprised three predictors (WM, age, and musical training) and different categories of long‐term memory for novel sequences LTM‐N (NT1, NT2, NT3, NT4). The table is organized in the following columns: measure of long‐term memory (“LTM”), predictors (“predictor”), regression coefficients (“β”), standard error (“SE”), *z*‐values (“z”), and *p*‐values (“p”).

## Data Availability

The data that support the findings of this study are available in the Supporting Information of this article.
